# Supplementary figures and images for: β-arrestin1 promotes tauopathy by transducing GPCR signaling, disrupting microtubules and autophagy
Source: Life Sci Alliance. 2021 Dec 3;5(3):e202101183. doi: 10.26508/lsa.202101183 (PMC8675912; doi:10.26508/lsa.202101183)

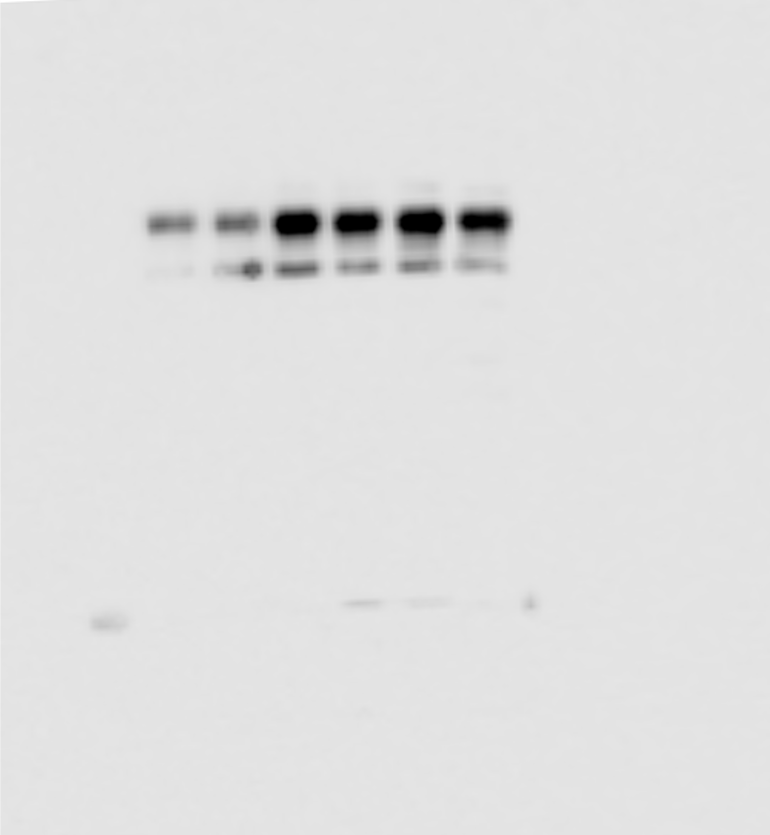

Supplement: Supplementary file 1 [file LSA-2021-01183_SdataF3.1.tif]

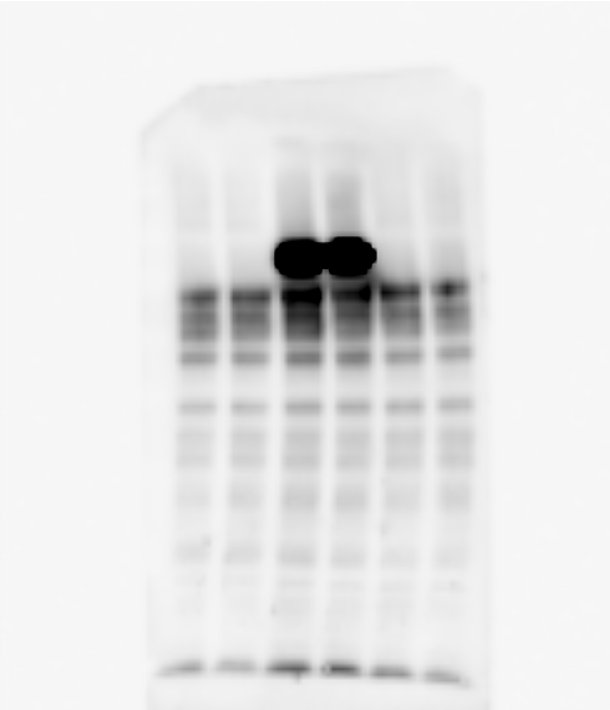

Supplement: Supplementary file 2 [file LSA-2021-01183_SdataF3.2.tif]

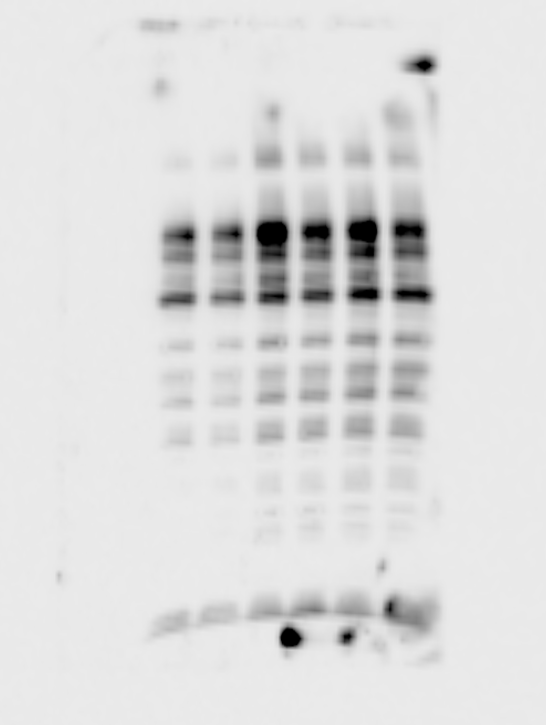

Supplement: Supplementary file 3 [file LSA-2021-01183_SdataF3.3.tif]

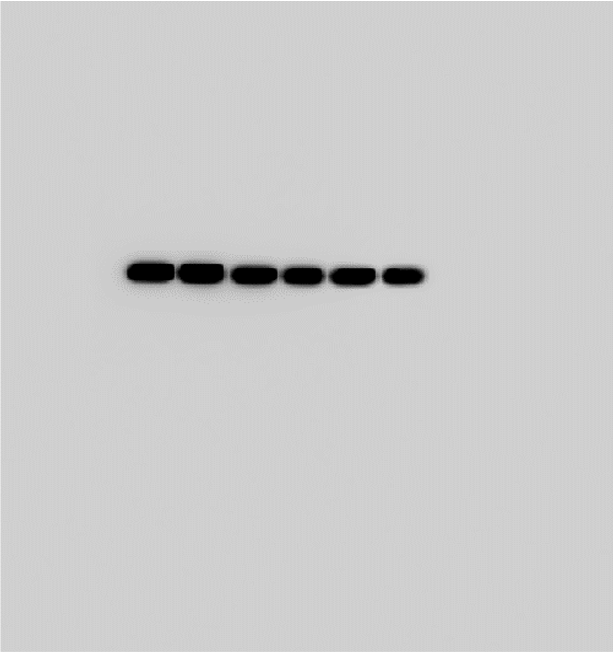

Supplement: Supplementary file 4 [file LSA-2021-01183_SdataF3.4.tif]
